# ESCC ATLAS: A population wide compendium of biomarkers for Esophageal Squamous Cell Carcinoma

**DOI:** 10.1038/s41598-018-30579-3

**Published:** 2018-08-24

**Authors:** Asna Tungekar, Sumana Mandarthi, Pooja Rajendra Mandaviya, Veerendra P. Gadekar, Ananthajith Tantry, Sowmya Kotian, Jyotshna Reddy, Divya Prabha, Sushma Bhat, Sweta Sahay, Roshan Mascarenhas, Raghavendra Rao Badkillaya, Manoj Kumar Nagasampige, Mohan Yelnadu, Harsh Pawar, Prashantha Hebbar, Manoj Kumar Kashyap

**Affiliations:** 1 Mbiomics, Manipal, Karnataka India; 2 Manipal Life Sciences Center, Manipal University, Manipal, Karnataka India; 3 Department of Biochemistry, Kasturba Medical College, Manipal University, Manipal, Karnataka India; 4 Manipal Center for Information Sciences, Manipal University, Manipal, Karnataka India; 5 Faculty of Applied Sciences and Biotechnology, Shoolini University of Biotechnology and Management Sciences, Bajhol, Solan, Himachal Pradesh 173229 India; 6 Infosys Technologies Ltd, Bangalore, Karnataka India; 7 Faculty of Biology, Technion-Israel Institute of Technology, Haifa, 3200003 Israel; 8 School of Life and Allied Health Sciences, Glocal University, Saharanpur, Uttar Pradesh 247001 India; 9 Newcastle University Medicine Malaysia, Johor Bahru, 79200 Malaysia; 10 Institute for Theoretical Chemistry, University of Vienna, Währingerstrasse 17, 1090 Vienna, Austria; 11 Department of Biotechnology, Alva’s college, Moodubidre, Karnataka India; 12 Department of Biotechnology, Sikkim Manipal University, Gangtok, Sikkim 737102 India

## Abstract

Esophageal cancer (EC) is the eighth most aggressive malignancy and its treatment remains a challenge due to the lack of biomarkers that can facilitate early detection. EC is identified in two major histological forms namely - Adenocarcinoma (EAC) and Squamous cell carcinoma (ESCC), each showing differences in the incidence among populations that are geographically separated. Hence the detection of potential drug target and biomarkers demands a population-centric understanding of the molecular and cellular mechanisms of EC. To provide an adequate impetus to the biomarker discovery for ESCC, which is the most prevalent esophageal cancer worldwide, here we have developed ESCC ATLAS, a manually curated database that integrates genetic, epigenetic, transcriptomic, and proteomic ESCC-related genes from the published literature. It consists of 3475 genes associated to molecular signatures such as, altered transcription (2600), altered translation (560), contain copy number variation/structural variations (233), SNPs (102), altered DNA methylation (82), Histone modifications (16) and miRNA based regulation (261). We provide a user-friendly web interface (http://www.esccatlas.org, freely accessible for academic, non-profit users) that facilitates the exploration and the analysis of genes among different populations. We anticipate it to be a valuable resource for the population specific investigation and biomarker discovery for ESCC.

## Introduction

Esophageal cancer (EC) is eighth most prevalent cancers worldwide with an estimate of 456,000 new cases in 2012, and the sixth most common cause of death from cancer eliciting 400,000 estimated cases^1^. Surprisingly in China alone, the estimated number of new EC cases and EC caused deaths are recorded as 291,238 and 218,957 respectively^2^. EC can be classified as esophageal adenocarcinoma (EAC) and esophageal squamous cell carcinoma (ESCC) according to the type of cells that are involved. EAC mainly occurs in the cells of mucus-secreting glands in the esophagus, whereas ESCC affects the thin cells that line the surface of the esophagus^3^. The incidence of EAC is predominant in the United States, whereas ESCC exhibits a greater geographical diversity in incidence, mortality and sex ratio especially between Eastern and Asian countries such as Turkey, Iran, Kazakhstan and especially northern and central China. More than half of global ESCC cases are recorded in China and accounts for 90% of the total EC cases alone^2,4–6^.

Some of the common and well-known risk factors associated with ESCC in Asian countries are tobacco/opium smoking, consumption of alcohol and chewing nass or areca nut often mixed with tobacco. In addition to these, certain regional food habits are also identified as the potential risk factors associated with the cause of ESCC *e.g*. consumption of beverages such as tea and coffee (linked to amount and temperature), consumption of nitrogenous component rich food, boiled yellow butter, and moldy cheese etc^7,8^. Most often these risk factors are strongly associated with sub-populations as they are linked to region-specific lifestyles. For example, although the use of tobacco and alcohol are clearly the major risk factors for developing ESCC, they are not accounted in Northern China (specifically Anyang region) because of the moderate alcohol consumption in this region^9^. Similarly, tobacco and opium smoking are recognized as the main cause for ESCC in Iranian population^10^, however, in Linxian, Shanxi province, China the dietary habits are suspected to be the main causal factor instead of smoking^11^. Several cohort studies have indicated that the obesity is positively associated with EAC, whereas negatively associated with ESCC in the European and USA population^12,13^. The increase in body mass index (BMI) is seen as a protective factor against ESCC in a Chinese cohort study^14^. This indicates for the existence of a regional difference in the risk factors for ESCC. Nevertheless, to date, there are no concrete studies available that can categorize the risk factors in respect to the regional population groups.

Apart from the lifestyles, genetic variations in the diverse population such as single nucleotide polymorphism (SNP)^15^, and structural variation^16^, along with epigenetic modifications such as DNA Methylation^17–19^, and Histone modification (HM)^20,21^, are also known to play a major role in ESCC. Due to the epidemiological differences for ESCC between the diversity of a population, there is an increased complexity in the selection of population-specific treatment. Hence, in order to get to the roots of ESCC, it becomes important to understand the population-specific cause of ESCC in the genomic and proteomic level.

Over the last decade, a number of studies are published on ESCC using genomics and proteomics high throughput techniques. Unfortunately, the generated data remain scattered in literature and unavailable as a compendium to the scientific community; An effort to compile this information in past resulted in a manually curated “Dragon database of genes involved in esophageal cancer (DDEC)^22^. However, DDEC is not updated recently. Focusing mainly on ESCC, here we have developed a new database called ‘ESCC ATLAS’ that catalogs all the genes and related molecular signatures that are involved in ESCC based on an exhaustive literature survey until May 2018. As of now the ESCC ATLAS contains 3475-curated gene from 403 unique publications from China (205), Japan (74), India (16), Korea (6), Iran (5), America (2), South Africa (4), Italy and Germany representing Europe (5) (Numbers in the bracket represents total publications from the corresponding region).

## Materials and Methods

### Data Collection and Screening

**Figure 1:**
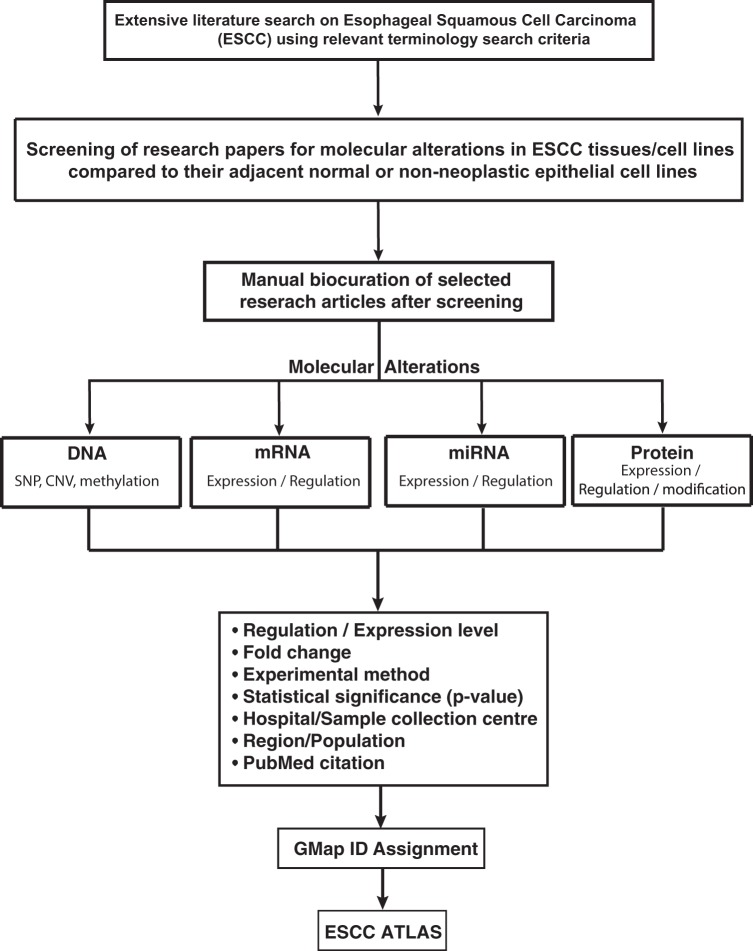
Schema for annotation of different types of molecular alterations in esophageal squamous cell carcinoma. The research articles published on esophageal squamous cell carcinoma (ESCC) are screened to filter the differentially expressed molecules at DNA, mRNA, miRNA and protein levels in ESCC tissues/cell lines as compared with their normal cell line or adjacent normal epithelia. The screened articles fulfilling the criteria described in the schema are manually curated to catalog the molecular alternations at DNA, mRNA, miRNA, and protein level. The information pertaining to ESCC and gene regulation status, the experimental approach used, analysis design, region of sample collection and along with the PubMed citation is provided for each molecule. The molecules are provided with external link to other database like OMIM, HPRD, HGNC and Ensembl to additional information about the molecule.

To ensure the highest quality in data collection process, all the entries in the ESCC ATLAS were manually collected based on a systematic search of the published literature in the PubMed/PubMed Central database. The keywords used to search for the relevant articles were designed considering the combination of the terminologies related to ESCC and its associated molecular alterations. The articles were considered eligible only if the reported clinical studies were conducted on human patients and if they included the (i) SNP, (ii) Copy number variation (iii) methylation, (iv) miRNA, (v) histone modifications, (vi) transcriptomic or (vii) proteomic regulation data (together referred as molecular signature hereafter) relevant to the etiology, pathophysiology of ESCC. To maintain the high standards of the collected data, special efforts were taken to catalog only those genes or relevant molecular signatures that were shown to have a significant association with ESCC by recording the reported statistical test values such as the *p-value* or fold change. Additionally, each entry in ESCC ATLAS was tagged with the cell line information (if reported), sample size, study method type and most importantly the population group name by making use of the reported location of the sample collection centers (hospital) or the reported population from which the study sample were collected. To fetch maximum information possible from each surveyed article, the submitted supplementary data were reviewed and included in ESCC ATLAS. Additionally, references cited in the articles were also considered for the data extraction when found relevant. The schema for the plan of annotation and biocuration has been shown in Fig. 1.

### Database Organization and Web Interface

The web application (front and back end) is developed using ExtJS (version 4.3), which facilitates efficient and quick multi-column search. To represent population specific data in the world map view, GoogleMap.js (ExtJS built-in plugin) was implemented. All the data in ESCC ATLAS were stored and managed using MySQL (version 5.0.51 b). The web services were built using Apache (version 2.2.17) and PHP (version 5.2.14) as server side scripting language. The whole system is hosted under Red Hat Enterprise Linux 5 environment.

### Population specific ESCC incidence and ESCC ATLAS entries

To get an overview of the worldwide population specific ESCC incidence, we collected the age-standardized rate of ESCC incidence available from GLOBOCAN 2012, and compared the number of collected molecular signatures for different populations in our survey.

### Functional enrichment analysis

Gene Ontology (GO) analysis was performed across all the three domains, Biological process, Molecular Function, and Cellular component. The populations analyzed were: Indian, Chinese, Japanese, Iranian, Korean and South African. The GO annotations associated to all 3475 genes from ESCC ATLAS were downloaded using biomaRt Bioconductor package in R (Ensembl release 89)^23^. An in-house R script was used to identify the overrepresented GO terms by comparing the proportion of genes annotated to a specific GO term in each population *(test gene group)* against the total protein-coding genes in human genome *(background gene group)*. The *prop.test()* function in R was implemented for the comparison, which calculates a chi-squared statistic to test the null hypothesis that the proportion of test and the background set of genes annotated to a specific GO terms are the same. Since multiple comparisons are performed in this analysis, the *p-values* thus obtained are adjusted to narrow down the chances of false discoveries using the False Discovery Rate method^24,25^ implemented in the *p.adjust* function in R. Additionally, for each of the GO term an enrichment score (ES) is calculated, where ES is, (Total number of annotated test genes/Total number of annotated background genes) * 100.

The overrepresented GO terms among the genes corresponding to each population were then selected based on the *p-value* cut off of <0.01 and summarized as scatterplots using REVIGO^26^. The enriched GO terms and their corresponding ES were supplied to the to REVIGO web server (http://revigo.irb.hr/). The option concerning semantic similarity measure was set to *SimRel* and the database for GO term size was set to Homo sapiens. The REVIGO checks for the redundancy of the GO terms represented by bubbles into a two-dimensional space by applying the multidimensional scaling to a matrix of GO terms semantic similarities.

### Protein-protein interactions

To get further insights on the important genes from the GO enrichment analysis, we looked for protein-protein interactions (PPI) networks using the PEPPER (Protein complex Expansion using Protein-Protein intERaction networks) application in Cytoscape^27^. PEPPER identifies the meaningful pathways/complexes as densely connected sub-networks from *seeds* (lists of input protein coding genes) using the information from protein interactions available in BioGRID database (in this analysis)^28^. PEPPER includes a topological and function-based post-processing pipeline for ranking the newly added proteins (*expansions*) according to their relevance to the seeds. The expansions are scored according to their co-occurrence with the *seeds* based on their impact on the overall connectivity of the subnetwork. The default parameter settings were used for the PEPPER algorithm. The output PPI network view were manually customized by hiding or showing the proteins/edges that were added by PEPPER algorithm to make it more readable in respect to the *seeds*. The interaction networks between the *expansions* are not shown.

### Statistical Analysis

The studies took into consideration for annotation purpose were considered only if a given molecule had either 2-fold up or downregulation, and/or differential regulation with *p* < 0.05. Since, the data included in the study is derived from the previously published studied by the scientist from all around the world, we applied these two-criteria only to avoid any tweaking of the data.

### “Ethics approval” and consent to participate

The study does not involved human, animal or cell lines as a material for experimental purpose. The data involved in the study is curated exclusively using published studies available through PubMed or PubMed Central (PMC) or Google search based research articles or sources.

## Results

### Data Summary

We presented the schema for collection of the data for this study in Fig. 1. We collected a total of 3475 non-redundant ESCC associated genes that fulfills the eligibility criteria to be included in ESCC ATLAS. The total number of published research articles referred for the data extraction for each molecular signature is shown in Dataset 1.

**Figure 2:**
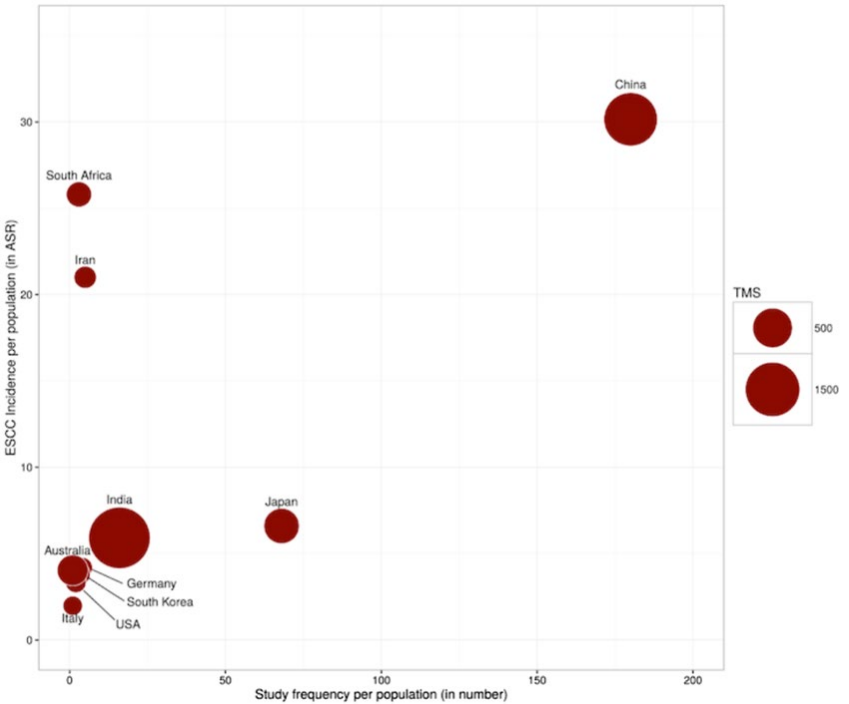
Population specific ESCC incidence and ESCC ATLAS entries. The observance of ESCC incidence was higher among Iranian and South African population, but the number of investigations focusing on these populations are very low. Hence, there are very few known ESCC associated molecular signatures. Here, *x-axis* indicates the total number of published articles focusing on specific population (that were surveyed and recorded in ESCC ATLAS) and the *y-axis* indicates the age-standardized rate (ASR) of ESCC incidence available from GLOBOCAN 2012. The size of scatter plots represents the total number of recorded molecular signatures to be associated with ESCC from the surveyed article.

Based on our literature survey and the ESCC incidence data available from GLOBOCAN 2012, we identified that despite of a very high incidence rate of ESCC among Iranian and South African population only few published studies are available focusing on these population that could aid the ESCC investigation and biomarker discovery (Fig. 2).

All the data in ESCC ATLAS is freely accessible via user-friendly web interface at http://www.esccatlas.org. Additionally, ESCC ATLAS also provides an option for the bulk data download as flat files which is useful for other downstream analysis.

### Collection of population specific ESCC associated molecular signatures

#### Genes involved in the transcriptomic and proteomic regulation of ESCC

**Figure 3:**
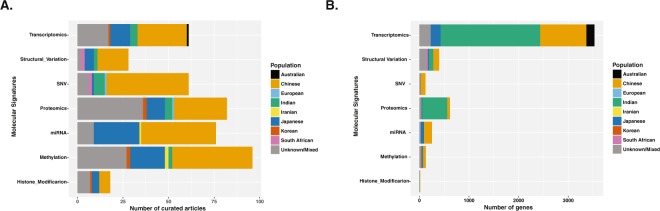
Distribution of molecular signatures vs. number of curated articles or number of genes across different populations. It shows statistics of (**A**) distribution of molecular signature (transcriptomics, proteomics, structural variation, SNV, miRNA, methylation, and histone modification) vs. number of curated research articles in different populations (coded with different colors) and (**B**) distribution of molecular signature (trasncriptomics, proteomics, structural variation, SNV, miRNA, methylation, and histone modification) vs. number of genes observed or curated from different populations (coded with different colors).

We found majority of the transcriptomic and proteomic signatures associated to ESCC were uniquely identified for different populations suggesting a population specific ESCC etiology that could be linked to different lifestyles and environmental exposures (Fig. 3A and B).

#### Transcriptome and proteome landscape of ESCC in global population

A total of 2592 unique transcriptomic signatures (genes) were curated. The expression status of these genes was studied and their distribution in different populations was analyzed. Gene regulation differences between populations with respect to the biological sample used to conduct the study was analyzed using transcriptomic data. It was found that the regulation remained consistent between populations in the same biological material. 17 genes were found where the biological material used differed, however, the regulation status remained consistent between populations. Out of these, only one gene, glutathione peroxidase 3 (GPX3) showed irregularities or difference in regulation between populations in different biological materials. GPX3 transcriptomic data was recorded for Chinese and Indian populations. Its expression was found to be downregulated in cell line samples of Chinese population, whereas in tissue samples of the same population it was found to be upregulated. Proteomic data was also analyzed to check for any inconsistencies with respect to biological material. For GPX3, expression regulation and status was found to be consistent between all population levels in proteomics in same or different biological materials.

An expression of *GPX3* was downregulated in ESCC as compared with normal samples^29,30^. Furthermore, GPX3 methylation was significantly higher in ESCC as compared to normal tissue samples^31^.

GPX3 was among an 8-gene signature panel that had been used for predicting the status (normal versus tumor) of gastric tissues with an accuracy of >96%. As an outcome of promoter hypermethylation, *GPX3* was down regulated in gastric cancer^32^. Furthermore, an *in vitro* study an association between GPX3 and lymph node metastasis was found in gastric cancer upon downregulation of GPX3 expression and promoter hypermethylation^33^.

In a previous study, *GPX3* was methylated in prostate cancer^34^ and an *in vitro* overexpression of GPX3 in prostate cancer cell lines could suppress formation of colonies as well as growth of cells in an anchorage-independent manner, and reduce the invasiness of the prostate cancer cells indicates the tumor suppressor activity of GPX3^35^. In case of breast cancer as well GPX3 was downregulated at mRNA as well as at protein levels in the inflammatory breast cancer as compared with non-IBC. Hypermethylation of GPX3 promoter was observed in breast cancer, but not in normal tissues^36^.

Population levels for transcriptomic data were found to be the following: Chinese, Japanese, and Indian. A total of 2060 genes in the Indian Population, 977 in the Chinese population, 203 genes in the Japanese population, and 163 ESCC-related genes were associated with the unknown/mixed population. A study of common and unique genes between combinations of different populations showed that the Chinese and Indian populations have the highest number of genes common between them. Only 1 gene (CSK2) was found to be common between all four populations, and was found to be upregulated.

Similarly, genes curated for proteomic data were also compared. Population levels were: Indian, Chinese and Japanese. The Indian population (293) had the highest number of genes curated, followed by Chinese (31) and Japanese (13). An analysis of common and unique genes between the populations revealed that only 3 genes (CDH1, EZR, YBX1) were found common between Indian and Chinese populations.

Among these genes E-cadherin (CDH1) is a tumor suppressor involved in epithelial cell-cell interactions. It’s a calcium-dependent cell adhesion protein involved in tumor invasiveness. *CDH1* has been reported to be hypermethylated in previous studies on ESCC^37–42^, and downregulation and/or loss of CDH1 has been documented in ESCC^40,41^. Interestingly, there was lack of association between -160C/A SNP of CDH1 and ESCC development^43^.

Ezrin is a cytoplasmic peripheral membranous protein, which is a member of the ezrin/radixin/moesin (ERM) family of proteins linking plasma membrane to F-actin cytoskeleton. Expression of EZR has been associated with invasiveness and lymph node metastasis of ESCC^44,45^. Translocation of EZR protein from plasma membrane to cytoplasm was observed in ESCC cells^46^. The knockdown inhibition of EZR led to decrease in growth, adhesion, and invasiveness of the ESCC cells *in vitro* and ability to induce tumorigenesis in *in vivo* conditions^47^.

Y-box binding protein 1 (YB-1 or YBX1) is an RNA and DNA binding factor. YBX1 expression had been observed not only in endothelial cells but in esophageal cancer cells as well. *YBX1* gene was upregulated and overexpressed in ESCC as compared with normal samples^48,49^. An elevated level of YBX1 protein associated with higher recurrence & lower survival in ESCC patients^49^.

Among another set of genes, we found two metalloproteinases (MMPs) genes (*MMP2 and MMP3*). These found in between Chinese and Japanese populations. These Zinc dependent MMPs including MMP2, MMP3, and MMP9 along with serine proteases play an important role in ESCC pathogenesis^50,51^. An overexpression of MMP2 has been reported in ESCC tissues as compared to adjacent normal epithelia^52^, and MMP3 SNP (MMP3 -1612 5A/6A) polymorphism was significantly associated with susceptibility to ESCC means ESCC subjects bearing 5A allele were more prone of getting ESCC as compared with 6A allele^50^.

There was not even a single found common between the Indian and Japanese populations, and same scenario was observed when we compared Indian, Chinese and Japanese populations.

**Figure 4:**
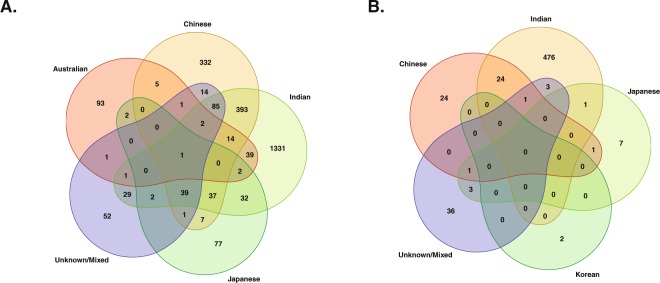
Distribution and overlapping of Transcriptomic and Proteomic data. Venn diagram representing distribution of (**A**) transcriptomic, and (**B**) proteomic evidences between different populations and indicating unique and common genes involved in etiology of ESCC between the different populations. We had only *CLDN4* gene with Transcriptomic evidences for Korean population, the same gene also found in Indian population. However, for better representation, we did not plot Korean population in (**A**).

Upon careful study of derived transcriptomic and proteomic population data, it is found that the highest number of recorded cases was found in the Indian population, followed by the Chinese and finally, the Japanese population. A list of complete and unique genes is provided in the supplementary material. The Venn diagram package from CRAN repository was used to create a graphical representation depicting common and unique gene distribution between different populations at the transcriptomic and proteomic levels (Fig. 4A and B).

#### SNPs in ESCC

In risk variants survey for ESCC, we found 85 genes harboring one or more risk variants in Chinese, 8 in Iranian, 5 in Indian, 4 in South African, 3 in European, and 1 in Japanese populations but none in Korean or unknown/mixed populations. Examining these population specific genes with that of transcriptome and proteome data, we could determine only 1 of the genes (*PADI4*) in Chinese (19 in transcriptome, 1 in proteome), but none in Japanese, Indian (4 in transcriptome), South Africa, European and Iranian populations that link with both altered transcriptomic and proteomic status. However, when we studied the transcriptomics and proteomics of global populations irrespective of population specificity in consideration, we could identify 8 genes: *GSTP1*, *PADI4*, *CCND1*, *FEN1*, *ADH1A*, *CRNN* and *S100A14* (40 in transcriptome; 11 in proteome) that were linked with both altered transcriptomic and proteomic status among 95 total unique genes harboring one or more risk variants (Supplementary Dataset # 2). Among these genes, PADI4 encode for protein arginine deiminase 4 catalyzes the hydrolytic deimination of arginine residues to produce citrulline and ammonia. Elevation of PADI4 was observed at mRNA as well as at protein levels in ESCC as well as in EAD^53^. It is an intracellular protein but it had been detected in pIasma samples of the cancer patients as well. In SNPs based study on PADI4, rs2240337 G > A SNP was found to be significantly associated with decreased risk of ESCC^54^. Earlier findings in SNPs study on PADI4 in EC reported rs10437048 and rs41265997 were significantly associated with decreased and increased risk of EC, respectively^53^.

#### Methylation in ESCC

We identified 51 genes with altered methylation status for Chinese (hyper: 50, hypo: 1), 22 for Japanese (hyper: 22, hypo: 0), 8 for Korean (hyper: 8, hypo: 0), 2 for Indian (hyper: 2, hypo: 0), and 2 for Iranian (hyper: 2, hypo: 0) populations. Examining these population specific genes (altered methylation) with that of transcriptome and proteome data, we could determine only 3 of the genes (*GADD45A*, *UPK1A*, and *C2orf40*) in Chinese (15 in transcriptome, 3 in proteome), 1 (TIMP3) in Japanese (3 in transcriptome, 1 in proteome), 1 (CLDN4) in Korean (4 in transcriptome, 1 in proteome), but none in Indian and Iranian populations that were linked with altered transcriptomic and proteomic status. However, when examined with transcriptomics and proteomics of global populations without specific populations into consideration, we could identify 16 of them (44 in transcriptome; 24 in proteome) were linked with both altered transcriptomic and proteomic status among 95 total unique methylated genes (hyper: 85; hypo: 4; unknown status: 6) (Dataset # 2).

#### Histone modification in ESCC

We could find 6 genes with altered histone modifications for Chinese (Acetylation 1; phosphorylation 6, deacetylation 1, gene PTK6 has both Deacetylation, Phosphorylation evidence), 4 for Japanese (Acetylation 3; phosphorylation 1, deacetylation 0), 1 for Korean (Acetylation 0; phosphorylation 1, deacetylation 0), and none for Indian and Iranian populations (no HM studies in these two). Examining these population specific genes (altered HM) with that of transcriptome and proteome data, we could determine only 3 of the genes in Chinese (0 in transcriptome, 3 in proteome), 1 in Japanese (0 in transcriptome, 1 in proteome), 1 in Korean (0 in transcriptome, 1 in proteome), link with altered proteomic status. However, none of the population specific gene could link to transcriptomic changes. Upon examining all the 18 unique HM altered genes (18; Acetylation 6; phosphorylation 11, deacetylation 2) with transcriptomics and proteomics of global populations without specific populations into consideration, we could identify 5 (PTK6, KLF4, MAGEA3, PIK3C2B, WDHD1) of them (5 in transcriptome; 16 in proteome) were linked with both altered transcriptomic and proteomic status (Dataset # 2).

#### Structural variation in ESCC

We found 92 (amplification: 35, Deletion: 6, LOH: 52) unique genes overlapping/published on the structural variations regions from so far published literature in Chinese, 91 (Amplification: 43, Deletion: 47, loss of heterozygosity i.e. LOH: 1) in Indian, 25 (Amplification: 22, Deletion: 13) in South African and 2 (Amplification: 1, LOH: 1) in Japanese populations. Examining these population specific genes with that of transcriptome and proteome data, we could determine only 4 of the genes (CCND1, CTTN, FAS, CDC25B) in Chinese (60 in transcriptome; 4 in proteome), 3 (20 in transcriptome; 3 in proteome) of the genes (GSN, KRT13, COL12A1) in Indian, but none in SA and Japanese populations, link with both altered transcriptomic and proteomic status. However, when examined with transcriptomics and proteomics of global populations without specific populations into consideration, we could identify 17 (*CCND1, CTTN, FAS, CDC25B, KRT13, COL12A1, GSR, IL1RN, PDCD4, TP63, SERPINH1, PSMA6, CFL1, GNPNAT1, GSTP1, PRKCI)* (Amplification: 12, Deletion:1, LOH: 4) of them were linked with both altered transcriptomic and proteomic status among 304 total unique genes published on structural variations so far (refer supplementary data).

#### miRs in ESCC

Each collected miRNA entries in ESCC ATLAS were mapped to their target genes by making use of the miRTarBase (a resource for experimentally validated miRNA targets) and www.microRNA.org (resource for predicted targets using miRanda algorithm). Currently, we have curated a total of 184 unique miRNAs in our database, out of which 61 miRNAs were mapped to their experimentally validated target genes listed in ESCC ATLAS. The miRNA entries were also checked for one to many mappings to their target genes, as a single miRNA can have more than one target gene(s). For example, from our curated list of miRNAs, hsa-miR-107, hsa-miR-326, hsa-miR-375 and hsa-miR-320a have multiple target genes.

A total of 184 miRNAs were studied, some of those were repetitive due to the redundancy of their respective target genes. The number of target genes for these cannot be accurately estimated due to the large number of target genes for a single miRNA species. The populations included in the study included Chinese, Japanese, and Iranian. We identified uniquely, 38 miRNA linked to the Chinese population, 25 in the Japanese Population, 1 in the Iranian and 2 in the unknown/mixed population. Upon comparing miRNA target gene regulation with that of transcriptomic and proteomic regulation data, we found two genes to show consistency in regulation status between miRNA transcriptomic and proteomic data. In the Chinese population, hsa-miR-451a was found to be downregulated. It’s target gene MMP-9 was found to be upregulated in the Chinese population, in both transcriptomic and proteomic datasets. Similarly, another gene FSCN1 had upregulated transcriptomic and proteomic status, is a target gene for hsa-miR-133b, which was found to be downregulated in the Chinese population. This corroborates the miRNA-gene-silencing mechanism, which is reflected in this analysis.

#### Population-wide GO analysis

**Figure 5:**
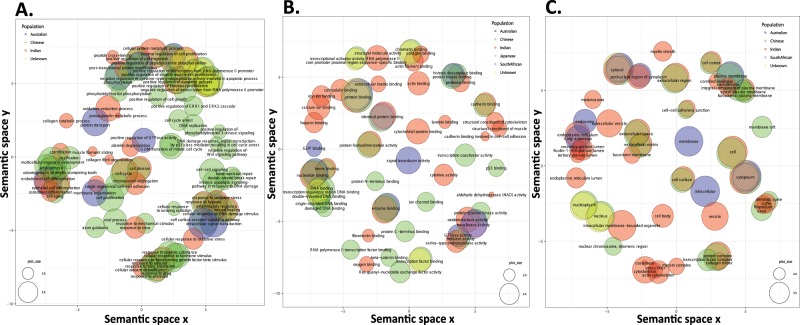
Data analysis for population specific overrepresented GO terms. Data analysis for population specific overrepresented GO terms corresponding to (**A**) biological process, (**B**) molecular function and (**C**) cellular component. In the scatterplot the semantically similar GO terms remain close together in cluster and are labeled with the representative GO term with the highest enrichment score. The bubbles corresponding to common GO terms between different populations are adjusted in two-dimensional space by adding or subtracting 0.15 semantic space units. The bubble size indicates the frequency of GO term in the underlying GOA database.

Several GO terms (with respect to Biological process, Molecular function and Cellular component) found through analysis, were common/overlapping and unique between populations such as Chinese, Indian, Japanese, South African and unknown/mixed (Fig. 5A,B and C). The list of all overrepresented GO terms and the number of genes associated to them are shown in the Dataset # 3.

Certain over-represented GO terms were found to overlap between populations (Fig. 5), for *e.g*. the GO terms “negative regulation of apoptosis”(11% of all test genes), “positive regulation of cell proliferation”(10% of all test genes) which are the hallmarks of cancer, were found to be over-represented in the Chinese, Indian and unknown/mixed populations.

**Figure 6:**
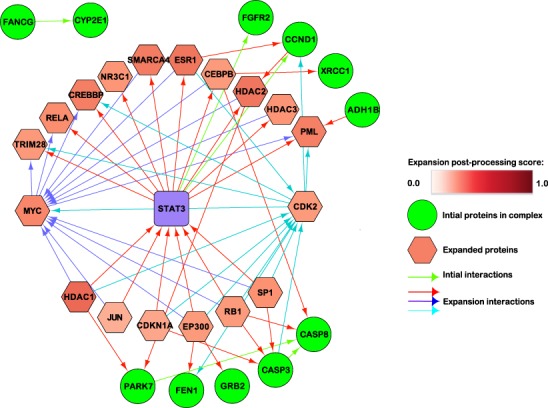
Protein-protein interactions. Hexagonal red nodes correspond to proteins that were added by PEPPER algorithm (*expansions*), the red shades of the hexagonal nodes correspond to the scores of relevance computed in the post-processing step. The purple square represents a protein of interest (*bait*, which is STAT3 in this case*)*. The rounded green nodes represent the list of input proteins (*preys*). The green edges represent the initial interactions between the *seeds (bait and preys)*. The red, dark and light blue edges are used to represent the interaction between the *seeds* and the *expansions*.

The GO term “positive regulation of cell proliferation” was found to exist in unknown/mixed, Chinese and Indian population. Among the test genes, we found that *STAT3, CCND1, FGFR2* were of significance when compared to their regulation status in our curated data. *FGFR2* was observed as overexpressed in the unknown/mixed population. In the Chinese population, STAT3 is commonly found overexpressed in ESCC and is associated with invasion of ESCCs^55^. Amplification and overexpression of CCND1 was found in ESCC in the North-eastern Chinese population^56^. Lastly, frequent gene amplification of FGFR2 is found in ESCC specimens^57^. This suggests that upregulation of STAT3, CCND1 and FGFR2 derails positive regulation of cell proliferation in ESCC. To get further insight into PPI network for STAT3, CCND1 and FGFR2, we used PEPPER to identify only few direct interactions within our input list of proteins (*seeds*, *purple and green nodes*) (Fig. 6). However the proteins (*expansions*) added by PEPPER algorithm constitutes a complex interaction network that connects together almost all of our input protein coding genes. The predicted network provides a global aspect for PPI of the key protein coding genes that could be involved in ESCC *e.g* STAT3 (Signal Transducer and Activator of Transcription 3), which is used as the *bait* protein in PEPPER PPI analysis clearly shows direct interactions between FGFR2 (Fibroblast growth factor receptors) and CCND1 (Cyclin D1). Indeed, oncogenic FGFR amplification is seen as a requirement that results in ectopic activation of the STAT3 transcriptional response^58^. And the constitutive activation of STAT3 and CCND1 overexpression is accounted for the proliferation, migration and invasion in gastric cancer cells^59^. Based on the interaction networks considering the expansion proteins we could observe STAT3 interacts with PML (Promyelocytic Leukemia Protein) and HDACs (Histone Deacetylase proteins). Indeed it is known that the acetylation of STAT3 and its subsequent binding with HDAC1 is involved in control of its nucleocytoplasmic distribution in Human hepatoblastoma HepG2 cells^60^. Similarly, PML is known to modulate interleukin (IL)-6-induced STAT3 activation and hepatoma cell growth by interacting with HDAC3^61^. RELA (NFκB) is known to be constitutively active in many cancers where it up-regulates anti-apoptotic and other oncogenic genes and here we observe a direct interaction between STAT3 and RELA. Studies have shown that STAT3 are involved in prolonging RELA nuclear retention through EP300 (acetyltransferase p300-mediated) RELA acetylation, thereby interfering with RELA nuclear export^62^. In summary, based on the PPI network analysis we obtained a global aspect of interaction networks between the candidate genes shortlisted genes from the GO enrichment analysis and other key protein-coding genes that could be involved in ESCC biogenesis.

Similarly, the GO term “negative regulation of apoptosis”, was found associated with the enriched term in the Chinese and the unknown/mixed population. It is known that CASP3 is involved in the apoptosis pathway and certain genetic variants of the gene confer susceptibility to ESCC. CASP3 829A > C (rs4647602) genotype is found to be associated with risk of ESCC. Variant rs4647602 (CC genotype) significantly reduced the transcriptional activity of caspase-3 and it was associated with an increased risk of ESCC in the Chinese population^57^. Furthermore, CASP3 is downregulated in the Chinese population when a comparison was made between normal versus basal cell hyperplasia^63^, but upregulated in ESCC as compared with adjacent normal tissues^64^.

Discernibly, some GO terms that may be of significance were unique for certain populations. For example, the term “cell differentiation” was uniquely associated with the unknown/mixed population. One of the genes, GRB2 that contributed to enrich the term, is found overexpressed with lymph node metastasis and poor prognosis in ESCC where cellular differentiation could be an influencing factor^65^. Evidently, from our curated data, we find that GRB2 is found to be upregulated in the unknown/mixed population.

Distinctly, the GO term “DNA repair” was found only in the Chinese population with 11% of test genes associated with this term. On comparing the genes for which this term was enriched, we found that some of them showed interesting expression levels in correlation with ESCC and literature. Curated SNP data shows that the polymorphisms found in the genes XRCC1, POLQ, FANCG, FEN1, SMUG1 were directly associated with risk of ESCC in the Chinese population^66,67^. These genes were also found in our GO analysis results for the term DNA repair. Expression status of some of these genes namely, XRCC1, FANCG and FEN1 was found to be upregulated in curated gene expression data. Therefore, one may infer a relation between polymorphisms in DNA repair genes that may lead to ESCC development and progression.

In the Indian population, 20% of the test genes were associated with the GO term “oxidation-reduction process”. The genes enriched for this term belong to family of cytochrome p450 enzymes (CYP1A1, CYP24A1, CYP26A1, CYP27B1, CYP2C18, CYP2C9, CYP2E1, CYP2J2, CYP3A4, CYP4B1, CYP4F12, CYP4F8, CYP4X1). Interestingly, all the genes found to be downregulated in the Indian population except CYP24A1, CYP26A1 and CYP27B1. Polymorphisms found in CYP2E1 and CYP1A1 are risk factors for development of ESCC^68,69^.

Another interesting observation found in the unknown/mixed population is that of oncogene DJ-1 (PARK7). Although out of 94 test genes, only *DJ-1* was associated with the GO term “negative regulation of TRAIL-activated signaling pathway”, it is found in our curated data and upregulated in the unknown/mixed population^70^ (Dataset # 2). The oncogene, *DJ-1* inhibits TRAIL-activated apoptosis pathway by blocking pro-caspase 8^71^. Incidentally, CASP8 was found to be downregulated in ESCC ATLAS in Chinese population. Overall, DJ1 plays an important role in transformation and ESCC progression, which suggests that DJ1 could be a prognostic marker for ESCC.

Alcohol consumption is widely known to be a risk factor for ESCC. In the Chinese population, GO analysis results showed enrichment for the terms “ethanol oxidation” and “alcohol metabolism”. Six of the test genes were associated with the former term and 3 of the test genes for the latter. Extensive literature review reveals that the alcohol metabolism pathway has two steps - ethanol oxidation, *i.e*. conversion of ethanol to acetaldehyde; and elimination of acetaldehyde from the body by conversion to acetate and finally to water and carbon dioxide. The first step carried out by involvement of two enzymes - Alcohol dehydrogenases (ADH1B) and cytochrome P450 (CYP2E1) enzymes. The second step is catalyzed by aldehyde dehydrogenase (ALDH2)^72^, it is found that variants of these genes affect the catalytic rate of the reaction in both steps^73^. An SNP found in the ADH1B gene (rs1229984) that replaces arginine with Histidine at the 48^th^ position makes the enzyme catalytically faster^72^. The heterozygous (ADH1B Arg/His) and homozygous (ADH1B His/His) variants of the gene possess higher catalytic activity and are faster in converting ethanol to acetaldehyde compared to the wild type (ADH1B Arg/Arg) allele. It is these variants that are associated with risk of ESCC. Acetaldehyde is a known carcinogen. It’s accumulation in the body due to its rapid production by the variant versions of the ADH1B enzyme leads to acetaldehyde carcinogenesis. This SNP is also found curated in our SNP dataset in the Chinese population, along with an SNP found in ALDH2 (rs671). The variant alleles of ALDH2 render it inactive, thereby promoting acetaldehyde accumulation and carcinogenesis in ESCC. With numerous examples of GO based analysis shows that ESCC ATLAS provided in-depth analysis on molecules differentially regulated between ESCC versus normal across different population.

Metabolism related aspects also contribute to malignancies including EC. In recent years, relations have been established between EC types and metabolism associated parameters such as body mass index, and blood pressure. A positive association was found between BMI and EAC, and a negatively association was found with risk of ESCC^12^. Obesity acts as a risk factor for BE as well as for EAC^74^. The underlying mechanisms behind association or role of obesity with ECs must be further explored in light of molecules such as leptin, adiponectin, estrogen and obesity related genes^75^. Interestingly adipose tissue has the ability to influences the development of tumor, which is dependent on secretor product adipokines and cytokines secreted by adipocytes and inflammatory cells, respectively^76^. An abundant supply of lipids by the adipocytes in tumor-microenvironment, supports progression and uncontrolled growth of the tumor^77^.

A good number of studies on single nucleotide polymorphisms (SNPs) based study was carried out on obesity related genes to find out an association with EAC, adenocarcinoma of esophagogastric junction, and ESCC, but there was no association found EGJAC or ESCC^78^. Three SNPs in NER pathway particularly the variant alleles of the XPD Lys751Gln, ERCC1 8092C/A and ERCC1 118C/T were associated with an increased risk of EAC^79^.

Overall, we found >600 common genes for Indian and Chinese population. The details of the genes have been provided in Dataset # 4. This large set of genes includes extracellular matrix related genes such as MMP1, MMP3, MPP7, MMP9, MMP10, MMP11, MMP12, MMP13, and MMP14. Some of the interesting genes include DUSP5 (Dual Specificity Phosphatase 5), which codes for DUSP5 protein. DUSP5 was found as downregulated in achalasia (a condition in which lack of relaxation of the lower esophageal sphincter occurs, act as a risk factor for ESCC, and 6% of the total achalasia subjects develop ESCC)^80^. Furthermore, DUSP5 was downregulated in ESCC as compared with adjacent normal in Indian and Chinese studies^30,81,82^. In addition to different molecules contributing to ESCC tumorigenesis in Indian and Chinese population, there is life style mainly diet pattern (Chinese and Indian particularly South Indians either prefers to have spicy food and/or consumption of pickled vegetables, and intake of alcohol and tobacco). In addition to these, to establish what are common factors among Chinese and India population, a comparative analysis is required to establish common pre-disposing and risk factors (achalasia, atrophic gastritis^83^, an infection by cytotoxin-associated gene A (CagA)-positive *Helicobacter pylori*^84^, injury to the esophagus, consumption of pickled vegetables, Plummer Vinson syndrome, Chaggas-associated mega-esophagus, and a history of certain head and neck cancers)^85^.

**Figure 7:**
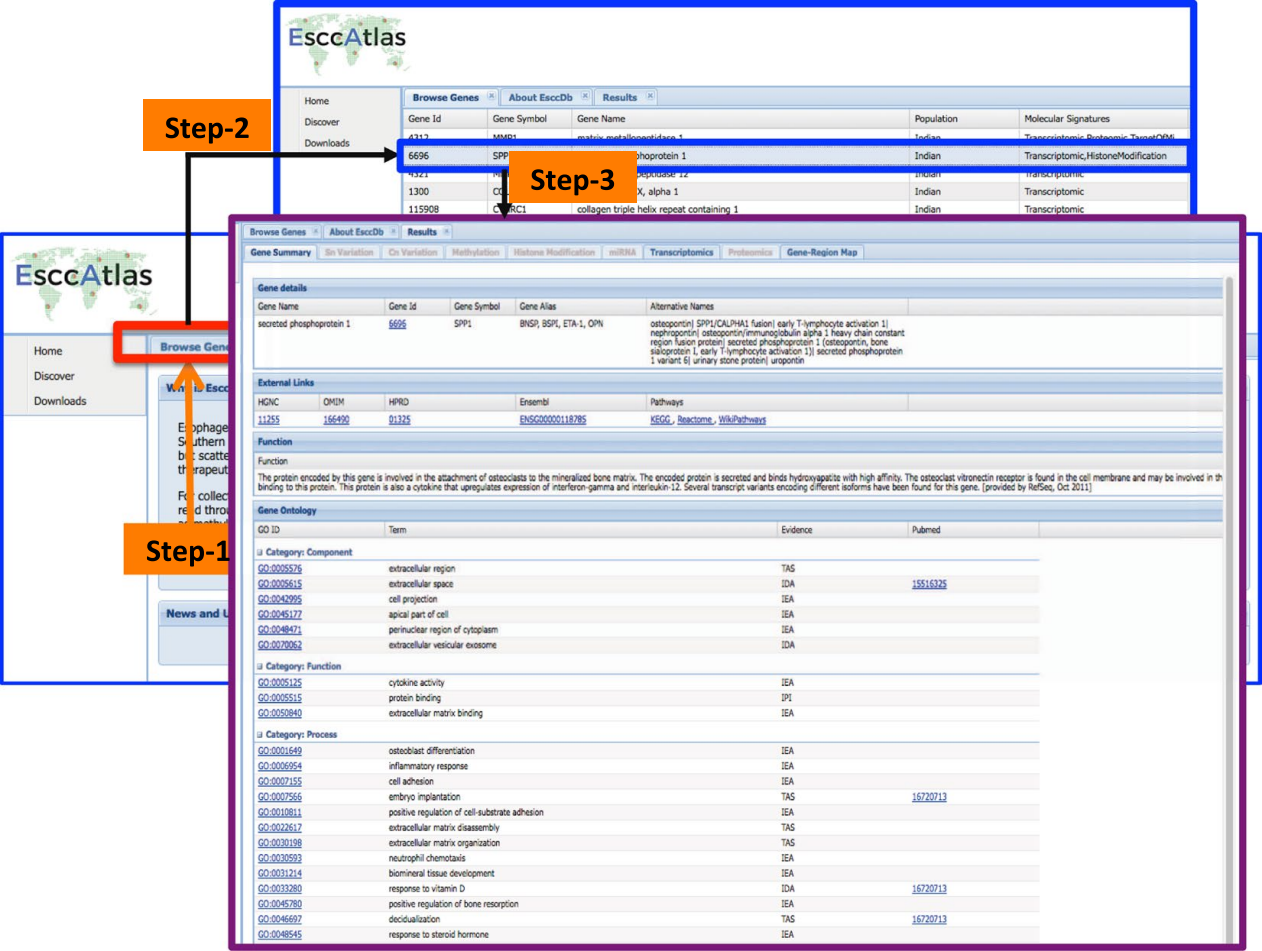
(**A**) screenshot of the primary information page for *OPN*/Osteopontin (gene/protein in ESCCDb. The query, browse and results tabs/pages for Osteopontin protein are shown (**B**) The molecule page for *OPN* with the DNA, mRNA, miRNA and protein level alterations, level of regulation, experimental approach used, PubMed citation and external links to publicly available resources. The figure was created using software Adobe Illustrator CS5 Version 15.0.0.

A quick search for the genes and their molecular signatures associated to ESCC in different population groups could be easily seen in 3 steps as shown in Fig. 7. Various useful external links are also embedded in the search results, for example, gene IDs, symbols, and gene alias from HUGO Gene Nomenclature Committee (HGNC), human protein reference database (HPRD) and Ensembl databases. The gene function and associated gene ontologies could also easily browsed with the links to GO database. A PubMed link to the reference research article is also made available. Further, upon entering the gene symbol for gene of interest e.g. osteopontin (*SPP1*) (Fig. 6), details about population specific, mRNA or protein level expression can be seen and additional information about the molecule is possible to explore by hitting external links like HGNC, HPRD or OMIM.

## Discussions

The “Omics” driven projects produced enormous amount of data. One of the biggest challenges faced by the scientific community was ‘how to handle and present this gigantic amount of data in a user friendly way’. In order to overcome, a number of cancer specific database developed including ONCOMINE (a cancer microarray database and web-based data-mining platform)^86^, pancreatic cancer^87–89^, Breast Cancer Database for breast cancer^90,91^, Dragon Database of Genes associated with Prostate Cancer^92^, Renal cancer gene database on renal cancer^93^, HLungDB: an integrated database of human lung cancer research^14^, Cervical Cancer Database on cervical cancer^94^, curatedOvarianDat on Clinically annotated data for the ovarian cancer transcriptome^95^, and Osteosarcoma, a database of osteosarcoma-associated genes^96^. Furthermore, there were efforts to make repository to have peptidome (a pool of peptides) detected in cancer-associated biofluids^97^.

In an earlier effort, DDEC was developed where a total number of 529 genes were reported. The database excluded the data derived from high-throughput microarray studies leaving an enormous amount of data not curated for ESCC^22^. Hence, with an aim of providing a central repository for scientific community in the area of ESCC, we developed ESCC ATLAS, a database system that focused on providing an in-depth resource of gene, miRNA and protein information and their relationships to ESCC overall and in a population specific manner. We made efforts to make ESCC ATLAS as a repository to have biocurated data for ESCC from low as well as high-throughput studies.

In the last 5 yrs, large amounts of data have been collected for making ESCC ATLAS. Information on ESCC data was obtained from the PubMed, PubMed central, google search engine. Genes, miRNAs, proteins, gene promoters, transcription factors, transcription factor-binding sites and the SNPs related to ESCC cancer have been collected and integrated into this system. The data collected in ESCC ATLAS was not only cataloged, but also exploited to gather regional disparity with ESCC research activities and molecular signatures discovered so far. Interestingly, cross-comparison of incidence of ESCC, number of molecular studies and total molecular signature identified between different populations suggests that despite high incidence of ESCC in Iranian and South African population low number of molecular studies lead to low number of molecular signatures identification. The 3475 genes/proteins in ESCC ATLAS are integrated in a way that investigator can rapidly query whether a gene or protein is found in human ESCC, and other detailed ESCC-related information about this gene. User-friendly query interfaces have made all the features of ESCC ATLAS easily accessible. ESCC ATLAS provides a comprehensive resource for human ESCC research. We believe that ESCC ATLAS will be particularly interesting to the life science community and will greatly facilitate cancer biologists’ mission of unraveling the pathogenesis of ESCC.

To extrapolate curated information in ESCC ATLAS, we created navigational links to other resources such as NCBI Entrez gene^98^, HGNC^99^, OMIM^100^, HPRD^101,102^, Ensemble^103^, KEGG^104^, WikiPathways^105^, GO^106^, miRBase^107^, and DGV^108^.

Strikingly, evaluation of transcriptome and proteome signatures among all populations suggested that from transcriptome only CSK2 found to be common with upregulation status in Chinese, Japanese, and Indian populations, whereas from proteome, genes CDH1, EZR, and YBX1 were found common between Indian and Chinese populations and genes MMP2, and MMP3 between Chinese and Japanese populations. This clearly exhibits, a significant number of population specific genes regulation involved in the etiology of ESCC between different populations.

Consolidation of GO analysis results with regulation of molecular signatures captured through our curation process, corroborate mechanism that drive progression of ESCC. The GO term “positive regulation of cell proliferation” (enriched from STAT3, CCND1, FGFR2) was found to exist in, Chinese and Indian population. Similarly, the GO term “negative regulation of apoptosis”, (enriched from CASP3) was found associated with Chinese population.

Distinctly, the term “cell differentiation” (GRB2) was uniquely associated with the unknown/mixed population, the term “DNA repair” (enriched from XRCC1, POLQ, FANCG, FEN1, SMUG1) was found only in the Chinese population, the GO term “oxidation-reduction process” (enriched from family of cytochrome p450 enzymes) were associated Indian population, and “negative regulation of TRAIL-activated signaling pathway” (enriched by oncogene DJ-1) in unknown/mixed population. Importantly, alcohol consumption is widely known to be a risk factor for ESCC, GO analysis results showed enrichment for the terms “ethanol oxidation” and “alcohol metabolism” in the Chinese population (found in Iranian as well).

Furthermore, ESCC ATLAS showed that variation between populations for a single molecule/gene are there as e.g. KRT17 gene is upregulated in 7 studies on Chinese (06) and North Indian (01) population, but downregulated in study on North-East Indian population^109^. The further collection data in ESCC ATLAS will allow researchers to study and compare the variation between the different populations. The information and link for the genes/proteins altered in ESCC are provided which allows researchers to know whether a particular molecule bears surface expression and secretory in nature to detect it in biological fluids using assays like ELISA assay.

Over the course of time, watching the approaches undertaken in human genomics, now research community is urging for an epoch with radical revision of human genomics^110^. In nutshell, we have biocurated the literature-derived data from studies on ESCC in ESCC ATLAS covering from DNA, mRNA, miRNA to protein levels. ESCC ATLAS provides scientific community with an easy access to different aspects of information regarding molecules driving ESCC carcinogenesis. In the future, we will incorporate more different kinds of genomics, proteomics, metabolomics, and pharmacogenomics based data in further updates to ESCC ATLAS, so that the database will continue to be an informative and valuable source ESCC associated molecular alterations and serve as a key repository for basic and translational research on ESCC.

## Conclusions

In conclusion, here we present ESCC ATLAS, an on-line database through which users can perform search for close to 3500 differentially regulated molecular alterations associated with ESCC tissues or cell lines. ESCC ATLAS is, as far as we know, the largest database on ESCC developed to be a user-friendly web based platform for the investigation of molecules to be queried either from the gene, miRNA or protein perspective. To make sure the relevance of ESCC ATLAS, we plan to update it annually to facilitate inclusion of new publicly available and annotation data. We strongly feel that ESCC ATLAS provides a novel and comprehensive tool for the systematic identification of molecular alterations in ESCC, which could be useful for the discovery of novel anticancer drugs targeting the ESCC, or for better understanding of the ESCC pathogenesis. All together, ESCC ATLAS is the largest database providing a comprehensive view for published data derived from either primary ESCC tissues samples or established ESCC cell lines. The data is provided in an easily available platform for the academic and not-for-profit organizations when exploring the molecular alterations that underlie ESCC. The current version of ESCC ATLAS covers DNA, mRNA, miRNA and protein level alterations. We will continue to review and assess the quality of our information and collection of ESCC-related molecular alterations in the future. For future, one of our goals is to identify the signal transduction pathway events that have significant impact on ESCC tumorigenesis and pathophysiology. In parallel, we will also catalog and include the downstream molecules of the altered signaling pathway in ESCC to fulfill the requirement towards identification of new drug targets, ESCC genes and novel mechanisms. For accelerating the research beyond geographic borders, between basic biomedical and clinical research in esophageal cancer, it is of utmost importance to have upcoming clinical and biological data on ESCC in a single repository like ESCC ATLAS. Hence we will invite researchers in the field of ESCC to deposit their data so that along with them, rest of the world also have access to the recent data on ESCC.

## Electronic supplementary material

Dataset 1

Dataset 2

Dataset 3

Dataset 4

## Data Availability

The data in ESCC ATLAS is freely accessible for academic Institutes/Universities and as well as for Not-for-profit organizations at the http://www.esccatlas.org website. All data generated and analyzed during our study are included in this published article and its supplementary information file-Dataset 1,2,3 and 4.
