# Supplementary material for: ESCC ATLAS: A population wide compendium of biomarkers for Esophageal Squamous Cell Carcinoma
Source: Sci Rep. 2018 Aug 24;8:12715. doi: 10.1038/s41598-018-30579-3 (PMC6109081; doi:10.1038/s41598-018-30579-3)
Supplement: Supplementary file 1 — Dataset 1 [file 41598_2018_30579_MOESM1_ESM.doc]

**Manuscript Title: ESCC ATLAS: A population wide compendium of biomarkers for Esophageal Squamous Cell Carcinoma**

**Author list:** Asna Tungekar, Sumana Mandarthi, Pooja Mandaviya, Veerendra P. Gadekar, Ananthajith Tantry, Sowmya Kotian, Jyotshna Reddy, Divya Prabha, Sushma Bhat, Sweta Sahay, Roshan Mascarenhas, Raghavendra Rao Badkillaya, Manoj Kumar Nagasampige, Mohan Yelnadu, Harsh Pawar, Prashantha Hebbar, Manoj K. Kashyap

**Dataset-1 Number of research articles surveyed (A) and total number of genes collected (G) for each molecular signatures in different population groups**

| **Population** | **SNP** | | **SV** | | **Methylation** | | | **HM** | | | **miRNA** | | | **Transcriptome** | | | **Proteome** | | |
| --- | --- | --- | --- | --- | --- | --- | --- | --- | --- | --- | --- | --- | --- | --- | --- | --- | --- | --- | --- |
|  | A | B | A | B | A | B | A | | B | A | | B | A | | B | A | | B |  |
| **American** | - | - | - | - | - | - | - | | - | 2 | | 1 | - | | - | - | | - |  |
| **Australian** | - | - | - | - | - | - | - | | - | -- | | - | 1 | | 162 | - | | - |  |
| **Chinese** | 46 | 95 | 16 | 92 | 43 | 51 | 6 | | 6 | 39 | | 157 | 28 | | 973 | 28 | | 37 |  |
| **European** | 4 | 3 | - | - | - | - | - | | - | - | | - | - | | - | 1 | | 1 |  |
| **Indian** | 5 | 5 | 2 | 91 | 2 | 2 | - | | - | - | | - | 4 | | 2022 | 3 | | 27 |  |
| **Iranian** | 2 | 9 | - | - | 2 | 2 | - | | - | 1 | | 1 | - | | - | - | | - |  |
| **Japanese** | 1 | 2 | 4 | 2 | 19 | 25 | 4 | | 4 | 25 | | 62 | 11 | | 199 | 10 | | 13 |  |
| **Korean** | - | - | - | - | 2 | 8 | 1 | | 1 | - | | - | 1 | | 1 | 2 | | 2 |  |
| **South African** | 2 | 8 | 2 | 35 | - | - | - | | - | - | | - | - | | - | - | | - |  |
| **without population** | 1 | 1 | 1 | 96 | 26 | 39 | 7 | | 7 | 7 | | 27 | 12 | | 314 | 25 | | 28 |  |

**A:** Number of research articles surveyed for each molecular signature

**B:** number of genes captured for each molecular signature

Numbers for abstract
Total unique genes: 3475 (includes 42 NOEntrezId genes)
Histone modification: 18 genes

SNP: 161 unique SNPs from 103 genes

Methylation: 95 unique genes with 88 hyper, 4 hypo (3 do not have meth status)

miRNA: 202 unique gene with 110 up-regulation, 56 with down, 28 with both up and down, 8 without reg status

CNV/structural variation: 304 unique genes with 185 amplification, 65 deletion, 52 LOH, 2 (both) amplification and  deletion

Transcriptome: unique genes 2705, upregulated 1259, downregulated 1371, both up and down 75

Proteome: unique genes 566, upregulated 311, downregulated 209, both up and down 46
